# Physical, Emotional, and Psychosocial Challenges Associated with Daily Dosing of HIV Medications and Their Impact on Indicators of Quality of Life: Findings from the Positive Perspectives Study

**DOI:** 10.1007/s10461-020-03055-1

**Published:** 2020-10-07

**Authors:** Patricia de los Rios, Chinyere Okoli, Erika Castellanos, Brent Allan, Benjamin Young, Garry Brough, Marvelous Muchenje, Anton Eremin, Giulio Maria Corbelli, Marta McBritton, W. David Hardy, Nicolas Van de Velde

**Affiliations:** 1 ViiV Healthcare, Quebec, Canada; 2 ViiV Healthcare, Brentford, Middlesex UK; 3 Global Action for Trans* Equality (GATE), Belize City, Belize; 4 International Council of AIDS Service Organizations (ICASO), Toronto, Canada; 5 ViiV Healthcare, Research Triangle Park, NC USA; 6 Positively UK, 345 City Road, London, UK; 7 AIDS Center Foundation, Moscow, Russia; 8 European AIDS Treatment Group, Rome, Lazio Italy; 9 Instituto Cultural Barong, São Paulo, Brazil; 10 Division of Infectious Diseases, Johns Hopkins University School of Medicine, Baltimore, MD USA

**Keywords:** Stigma, Difficulty swallowing, Quality of life, Privacy, Antiretrovirals

## Abstract

To assess challenges with daily oral antiretroviral therapy (ART), we analyzed data for 2389 participants in the 2019 Positive Perspectives survey of people living with HIV in 25 countries. ART-related challenges reported included difficulty swallowing pills (33.1% [790/2389]); stress from daily dosing routine (33.3% [795/2389]); bad memories from daily intake of HIV medication (35.1%[839/2389]), and concern “that having to take pills every day means a greater chance of revealing my HIV status to others” (37.9% [906/2389]). Individuals who felt empowered by daily oral dosing [“taking my pill(s) every day reassures me that my HIV is being kept under control”] had 69% higher odds of optimal overall health (AOR 1.69, 95% CI 1.40–2.04). Conversely, odds of optimal overall health were lower among those who felt daily pill intake “limits my day-to-day life” (AOR 0.53, 95% CI 0.44–0.64). These findings show that there is need for increased flexibility of ART delivery to meet diverse patient needs.

**Electronic supplementary material:**

The online version of this article (10.1007/s10461-020-03055-1) contains supplementary material, which is available to authorized users.

## Introduction

Globally, there are an estimated 37.9 million people living with HIV/AIDS (PLHIV) [1]. PLHIV report a myriad of emotional and psychosocial problems such as coping with being diagnosed with the disease and acceptance of it, sharing their diagnosis with others, decision making regarding treatment, sexual activity and desires of parenthood, seeking social/emotional support, and workplace-related challenges [2–8]. Substance abuse as a coping mechanism may further worsen psychological and mental health problems among PLHIV [9–11].

Stigma among PLHIV significantly causes bitter feelings, shame, embarrassment, hopelessness, isolation, and reluctance on the part of some PLHIV to access healthcare [12–14]. Different socio-cognitive structures of stigma exist, including stereotypes, prejudices, and discrimination; these could be experienced by PLHIV as internalized, anticipated, or enacted stigma [12–14]. Stereotyping, where PLHIV are perceived to have certain stigmatizing attributes, can be particularly aggravating, especially if the individual does not identify with one or more of the stigmatized statuses [13]. To avoid discrimination, some may choose not to share their HIV status [15, 16], especially if they have witnessed other PLHIV at the receiving end of stigma [13]. Unfortunately, this may come at the cost of receiving fragmented healthcare or failing to get needed social support from friends and family. Other effects of stigma among PLHIV could include low self-worth, constricted social networks, and depression [15–21].

Besides stigma, PLHIV also face unique treatment needs [22]; ART influences quality of life in ways that are complex and multidimensional. On the one hand, PLHIV who have achieved and maintained undetectable viral loads cannot transmit the virus to their sexual partner [23], which can improve their quality of life and self-esteem. However, PLHIV may experience pill fatigue with daily regimens [24, 25]; experience of side effects could also impact negatively on quality of life. More so, individuals with gastro-intestinal conditions may poorly tolerate oral medicines while memory disorders may make it challenging for PLHIV to remember taking their medicines [26–28]. The emotional challenge of daily dosing, inconvenient scheduling, and lifestyle changes due to HIV treatment can be disruptive to PLHIV’s lives [29]. Because of these medication challenges, HIV and its treatment may redefine the daily lives of PLHIV in ways that are profound and consequential, including their interactions with others, their motivation to work, and their overall outlook on life.

Through the years, the worldview regarding health and wellness has evolved from being “diagnosis-focused” to more “person-focused”, greater emphasis is now placed on the individual’s positive psychological function [30]. The World Health Organization’s definition of health similarly goes beyond ensuring “absence of disease or infirmity”, rather, it focuses on promoting positive health-related quality of life including optimal physical, mental, and social wellbeing [31]. These ideals are aptly captured in the fourth ‘90′ target for HIV care, which seeks to improve quality of life among PLHIV [32]. In line with this, holistic care of PLHIV involves a consideration of the breadth of challenges faced by PLHIV—issues that may be a constant fixture in their lives even if they are virally suppressed [33, 34]. Some of the social and emotional challenges faced by PLHIV may be caused by, associated with, or exacerbated because of HIV, and therefore require deliberate consideration as part of patient-centered care. Information on the full spectrum of challenges experienced by PLHIV can facilitate holistic care. For example, it may highlight the need for various providers, including medical and mental health providers, to utilize a team approach and have open communication to prevent fragmented care.

Given the diversity of experiences, perspectives, and realities of HIV over time—across ages, countries, and backgrounds, there is need for data to better understand the extent of physical, emotional, and psychosocial challenges faced by PLHIV. This study therefore investigated treatment and non-treatment challenges among PLHIV, including medical conditions (e.g., difficulty swallowing, mental health disorders, insomnia, substance use disorder); emotional challenges (e.g., pill fatigue); and stigma. We quantified the percentage of PLHIV reporting these various challenges and explored the relationship with indicators of health-related quality of life. A better understanding of these issues is important because it would be difficult to address suboptimal adherence or other poor health outcomes without first understanding and addressing their root causes.

## Methods

### Data Source

Data were obtained from the second wave of the Positive Perspectives Study, a cross-sectional survey of 2389 adults aged 18 years or older with positive diagnosis of HIV 1. We have previously reported on the study methodology as well as research findings in other areas such as polypharmacy, suboptimal adherence, patient-provider engagement, and aspirations towards improved treatment [35–39]. In brief, participants were recruited from 25 countries using non-probability sampling approaches and surveys were completed online. Participating countries were Argentina, Australia, Austria, Belgium, Brazil, Canada, Chile, China, France, Germany, Ireland, Italy, Japan, Mexico, Netherlands, Poland, Portugal, Russia, South Africa, Spain, Switzerland, Taiwan, United Kingdom, South Korea, and USA. Our analyses were performed on the pooled sample of 2389 PLHIV.

### Ethical Review

Ethical review was done by the Pearl Institutional Review Board (no. 18–080622, blanket review for all study sites) as well as from the Sefako Makgatho Research Ethics Committee (no. SMUREC/M/223/2019, review for South Africa specifically).

### Measures

#### Physical, Emotional, and Psychosocial Challenges to Treatment

Self-reported diagnosis of several medical conditions was assessed; participants were told to “Please select which medical conditions below you have ever been diagnosed with by a doctor or other healthcare professional.” Difficulty swallowing pills was self-reported and was defined as scores ≥ 3 (on an ordinal scale of increasing difficulty from 1 to 5) in response to the question: "In general, how easy or difficult do you find it to swallow pills?".

Participants were asked if they were in agreement (“Agree”/“Strongly agree” vs. “Disagree”/“Strongly disagree”/“Neither agree nor disagree”) with the following nine statements: “Taking my pill(s) every day reassures me that my HIV is being kept under control”; “I have no problem managing the pill(s) I need to take each day for my HIV”; “Having to remember to take my HIV medication every day causes me stress or anxiety”; “Taking my HIV medication limits my day-to-day life”; “Taking pills for HIV every day is a daily reminder of HIV in my life”; “Taking pills for HIV every day is a link to some bad memories from my past”; “I worry about forgetting to take my daily HIV medication or taking it later than planned”; “I worry that having to take pills every day means a greater chance of revealing my HIV status to others”; “As long as my HIV stays suppressed, I would prefer not having to take HIV medication every day”. These variables, which captured a wide spectrum of attitudes—both favorable and unfavorable regarding daily oral ART—were analyzed to determine how PLHIV perceptions and acceptance of treatment influenced self-reported health outcomes that could be considered markers of health-related quality of life.

The survey further assessed whether participants had, within the past 6 months, “ever hidden or disguised [their] HIV medication to avoid revealing [their] status”, with whom they had shared their HIV status, and reasons for which they did not share their “HIV status with someone in the past”.

#### Clinical and Demographic Parameters

Self-reported virologic control was defined as a response of “‘Undetectable’ or ‘Suppressed’” (vs. “‘Detectable’ or ‘Unsuppressed’”, “I don’t know”; or “Prefer not to say”) to the question “What is your most recent viral load? Self-rated health was dichotomized as optimal (“Good”, or “Very good”) vs suboptimal (“Very poor”, “Poor”, “Neither good nor poor”). Participants indicated the reason(s) for which they missed ART within the past month, if any, and for each reason, the number of times they missed.

Participants were also asked whether they felt there was room for improvement with their HIV medication and with their overall HIV management. A poor outlook regarding HIV-related mortality was an affirmative response to either of the following, “HIV will reduce my life span”, or “Because of my HIV, I do not plan for my old age”. Other clinical parameters included presence of non-HIV co-morbidities (none, one only, or ≥ two), and year of HIV diagnosis (pre-2010, 2011–2016, or 2017–2019). Demographic characteristics included age, gender, sexual orientation, geographic region, domicile, and employment.

### Analyses

Prevalence estimates were calculated in descriptive analyses to characterize the range of emotional, psychological, and physical challenges faced by PLHIV in relation to daily oral ART. Trends in selected outcomes by duration of disease (time since HIV diagnosis) were analyzed using logistic regression, adjusting for gender, geographic region, education, and ethnicity. In addition, we used logistic regression to measure the relationship between different emotional, psychological, and physical challenges and self-reported health outcomes (self-rated overall health, perceived unmet treatment needs, and self-reported virologic suppression) adjusting for age, gender, ethnicity, education, region, and duration of disease.

To better understand the underlying reasons for privacy concerns, we compared reasons for not sharing HIV status between those who ever vs. never hid/disguised their HIV medicines. We further used multivariable logistic regression analyses to examine how specific concerns regarding sharing of HIV status were related to whom PLHIV shared their status with, controlling for age, gender, ethnicity, education, region, and duration of disease. Results were deemed statistically significant at p < 0.05. All analyses were performed with R Version 3.6.1.

## Results

Of the 2389 study participants, mean age was 41.2 years (standard deviation = 12.2), while median duration of HIV was 10.1 years (standard deviation = 9.6). Self-reported ethnicity was as follows among all participants, regardless of their nationality: Asian, 3.7% (88/2389), Hispanic/Latinx, 1.5% (36/2389), black, 11.5% (275/2389), multiracial, 9.3% (222/2389), white, 58.3% (1393/2389), and unknown ethnicity, 15.7% (375/2389). By sexual orientation, 41.2% were heterosexual, 45.8% were homosexual, while 13.0% identified as bisexual/asexual/pansexual/other sexual orientation. By gender, 67.9% were men, 29.1% were women, while 2.9% identified as nonbinary/other gender. By year of HIV diagnosis, 22.9% were diagnosed during 2017–2019, 38.2% during 2010–2016, while 38.8% were diagnosed prior to 2010. A variety of physical, emotional, and psychosocial challenges were reported by PLHIV, as shown in Table 1.

**Table 1:** Percentage of people living with HIV aged ≥ 18 years from 25 countries who reported various challenges with their treatment, 2019 (N = 2389)

Characteristic	Category	N	Difficulty swallowing pills	Stressed/anxious about their daily dosing schedule	Perception HIV medication limits day-to-day life	Taking HIV pills every day is a daily reminder of HIV	Taking HIV pills everyday cues bad memories	Worry that taking HIV medicines everyday increases chance of revealing HIV status
Total	Overall	2389	33.1	33.3	28.9	58.4	35.1	37.9
Physical health	Suboptimal	953	44.3	40.1	37.5	61.6	42.4	43.3
	Optimal	1436	25.6	28.8	23.3	56.2	30.3	34.3
Mental health	Suboptimal	1013	45.9	42.9	37.0	64.5	45.2	45.0
	Optimal	1376	23.6	26.2	23.0	53.9	27.7	32.7
Sexual health	Suboptimal	1227	40.1	36.7	33.7	61.9	41.6	41.0
	Optimal	1162	25.6	29.7	23.8	54.6	28.3	34.7
Overall health	Suboptimal	1012	42.7	41.9	36.9	65.3	43.7	43.9
	Optimal	1377	26.0	26.9	23.1	53.2	28.8	33.6
Gender	Men	1623	31.5	30.7	27.2	56.6	32.3	35.7
	Other	70	34.3	40.0	42.9	74.3	50.0	55.7
	Women	696	36.5	38.5	31.6	60.8	40.2	41.4
Sexual orientation	Heterosexual	984	46.0	37.5	34.2	58.0	39.9	43.2
	Homosexual	1094	22.0	26.9	21.1	56.2	29.3	30.8
	Other	311	30.9	42.4	39.5	66.9	40.5	46.3
Age, years	< 50	1690	37.1	38.3	32.8	60.6	37.7	43.3
	50+	699	23.3	21.0	19.6	52.9	28.9	24.9
HIV diagnosis year	2017–19	548	40.9	42.7	37.6	67.7	45.1	52.7
	2010–16	913	35.5	37.9	33.4	59.5	36.4	43.5
	Pre-2010	928	26.1	23.2	19.4	51.7	28.0	23.7
Geographic Region^a^	Northern America	520	51.5	39.4	39.4	63.8	41.7	42.1
	Europe	1119	25.2	29.5	24.9	55.7	32.3	33.3
	Latin America	221	26.7	38.5	25.3	72.4	40.3	42.1
	Asia	230	43.5	49.1	44.3	53.9	42.2	57.0
	Australia	120	15.0	18.3	10.8	53.3	26.7	19.2
	South Africa	179	35.2	22.3	20.1	50.8	24.0	37.4
Domicile	Metropolitan	1335	27.2	33.6	26.7	59.9	33.9	36.6
	Non-metropolitan	1054	40.5	32.8	31.8	56.5	36.7	39.7
Employment	Employed	1653	32.5	33.8	29.0	58.4	35.6	39.5
	Nonemployed	736	34.4	32.1	28.7	58.2	34.1	34.4
Non-HIV comorbidities	None (HIV only)	993	36.5	38.6	33.6	59.1	38.7	44.8
	One only	470	32.1	28.5	25.3	56.2	29.1	40.2
	2 +comorbidities	926	29.9	30.0	25.7	58.6	34.3	29.4
ART side effects	None reported	1348	27.5	23.6	17.6	49.9	26.1	28.5
	Non-gastrointestinal only	356	30.9	35.7	33.7	61.8	35.7	44.1
	Gastrointestinal	685	45.1	51.1	48.8	73.3	52.6	53.3

*ART* antiretroviral therapy

^a^Countries by region were: Northern America (U.S. and Canada); Europe (Austria, Belgium, France, Germany, Italy, the Netherlands, Poland, Portugal, Ireland, Russia, Spain, Switzerland, and the UK); Latin America (Argentina, Brazil, Chile, and Mexico), Asia (China, Japan, South Korea, and Taiwan)

### Physical Challenges

**Fig. 1:**
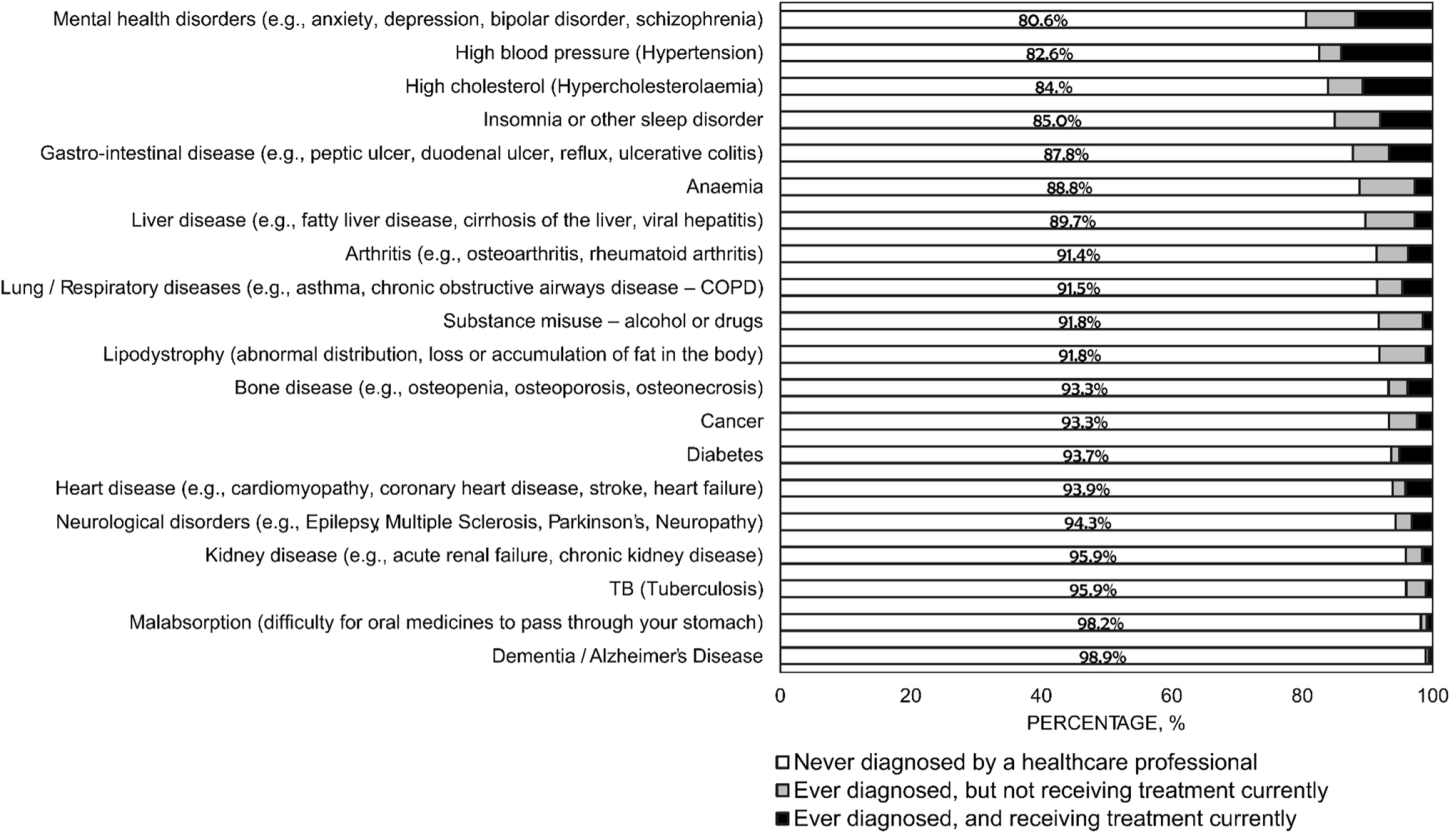
Percentage of people living with HIV in 25 countries who reported being diagnosed of various health conditions by a healthcare provider, 2019 (N = 2389)

Self-reported diagnosis of various medical conditions within the overall population are depicted in Fig. 1, and included the following conditions that have potential to interfere with daily oral administration of ART: dementia/Alzheimer’s disease (1.1%, 26/2389), malabsorption (1.8%, 43/2389); neurological disorders (5.7%, 136/2389), substance disorder (8.3%, 197/2389), gastrointestinal conditions (12.2%, 292/2389), and insomnia (15.0%, 358/2389). Of all study participants, 33.1% (790/2389) reported difficulty swallowing pills. The percentage of PLHIV who missed ART ≥ 1 time in the past month because of “trouble swallowing pills” was significantly higher among those reporting “difficulty swallowing pills” (44.4%, 351/790) vs those not reporting difficulty swallowing (9.5%, 152/1599) (p < 0.001). The percentage who missed ART ≥ 1 time in the past month because they “had a problem taking pills at a specific time (with meals, on empty stomach, etc.)” was significantly higher among those reporting malabsorption (74.4%, 32/43) vs. those without a report of malabsorption (30.3%, 710/2346) (p < 0.001). The percentage who missed ART ≥ 1 time in the past month because they “used recreational drugs” was significantly higher among those with vs. without a self-reported diagnosis of substance misuse (29.4% [58/197] vs. 22.7% [498/2192], p = 0.032).

Overall, 43.6% (1041/2389) of participants experienced side effects with their HIV medications, and of these 65.8% (685/1041) reported experiencing “gastro-intestinal side effects (stomach upset, stomachache/pain, diarrhoea)”. Of those experiencing any side effects, 71.6% indicated that “the side effects of my HIV medication impact my daily life”. The percentage who missed ART ≥ 1 time in the past month because they “wanted to avoid side effects” was significantly higher among those experiencing any side effects with their current HIV medications than those without any report of side effects (35.2% [366/1041] vs. 16.8% [226/1348], p < 0.001).

**Fig. 2:**
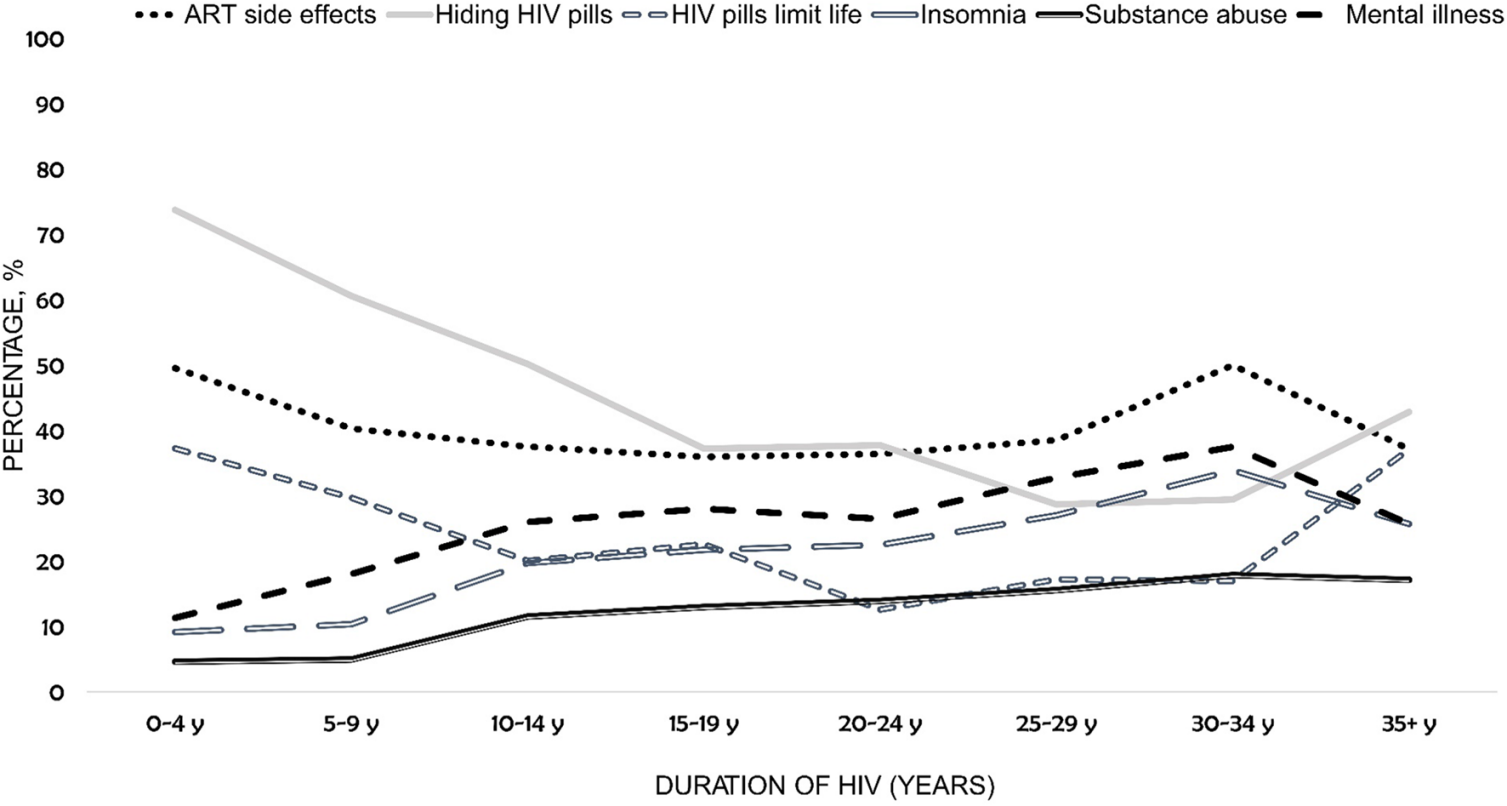
Percentage who reported substance use disorder, ART side effects, HIV-related privacy concerns, and emotional challenges, by time since diagnosis, among people living with HIV in 25 countries, 2019 (N = 2389). *ART* antiretroviral therapy. All trends were statistically significant (p-trend < 0.05). Assessment of whether observed trends were statistically significant was performed in a binary logistic regression model, adjusted for gender, geographic region, education, and ethnicity. The following indicators saw a statistically significant downward trend by duration of disease: experience of ART side effects, hiding of HIV pills, and the perception that pills limit life. All other depicted outcomes increased significantly at p < 0.05

**Fig. 3:**
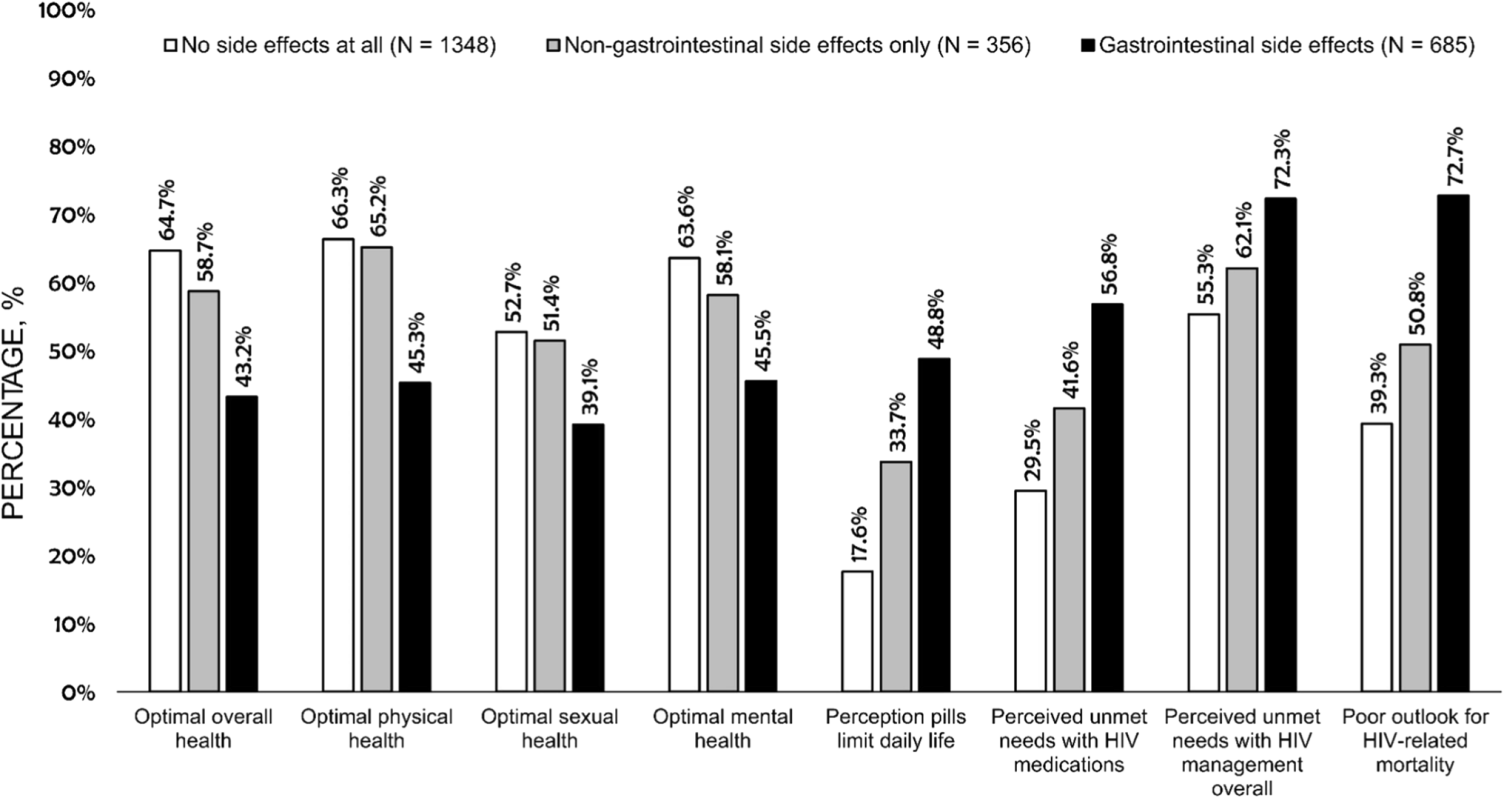
Comparisons of percentages for self-rated health and various treatment-related outcomes, by reported ART side effects, among people living with HIV from 25 countries, 2019 (N = 2389). *ART* antiretroviral therapy. All p < 0.001 based on χ^2^ tests. "Optimal” self-rated health was assessed within the past 4 weeks; self-rating of health as "good" or "very good" was classified as "optimal" (vs. "neither good nor poor", "poor", or "very poor"). Participants who answered “Agree” or “Strongly agree” to the statement “Taking my HIV medication limits my day-to-day life” were classified as perceiving that daily intake of HIV pills limited their daily life. The perception that there was room for improving current HIV medication was defined as a score of ≥ 4 (on an ordinal scale from 1 to 5) in response to the question "Do you feel that there is room for improvement with your current HIV medication or do you feel that it fully meets your needs?" The feeling that "there is room for improving the way my HIV is managed" was based on a response of "Agree" or "Strongly agree" to this statement. Poor self-prognosis regarding HIV mortality was an affirmative response ("Agree" or "Strongly agree") to either statement below: "HIV will reduce my life span" or "Because of my HIV, I do not plan for my old age"

The percentage of PLHIV reporting any side effects from their “current HIV medication” decreased significantly with increasing duration of HIV, from 49.6% (504/1017) among those diagnosed 0–4 years ago, to 37.1% (13/35) among those living with HIV for ≥ 35 years (p-trend = 0.005, adjusted for gender, geographic region, education, and ethnicity, Fig. 2). Conversely, the prevalence of self-reported substance use disorder increased with increasing duration of HIV, from 4.6% (47/1017) among those diagnosed 0–4 years ago, to 17.1% (6/35) among those living with HIV for ≥ 35 years (adjusted p-trend < 0.0001); this upward trend in substance misuse by increasing time since HIV diagnosis remained significant even after adjusting for age. Individuals experiencing gastrointestinal side effects and those with difficulty swallowing consistently had the poorest health outcomes within both crude and adjusted analyses (Fig. 3, Table 2).

**Table 2:** Adjusted odds ratios with 95% confidence intervals for the associations between physical, emotional, and psychosocial challenges and self-rated health as well as various treatment-related outcomes among people living with HIV from 25 countries, 2019 (N = 2389)

Indicator	Category	Optimal overall health^a^	Perception HIV medication can be improved^b^	Perception HIV management can be improved^c^	Poor outlook on HIV mortality^d^
Daily pills reassure me my HIV is controlled	Reported vs not reported	1.69 (1.40–2.04)*	0.88 (0.72–1.07)	1.39 (1.15–1.69)*	0.75 (0.62–0.91)*
I have no problem managing daily pills	Reported vs not reported	1.87 (1.57–2.23)*	0.77 (0.64–0.92)*	1.09 (0.91–1.31)	0.73 (0.61–0.87)*
Daily pills cause me stress or anxiety	Reported vs not reported	0.52 (0.43–0.62)*	2.34 (1.95–2.81)*	1.59 (1.32–1.92)*	3.3 (2.73–4.00)*
HIV medication limits my day-to-day life	Reported vs not reported	0.53 (0.44–0.64)*	2.35 (1.95–2.84)*	1.89 (1.55–2.31)*	4.34 (3.53–5.34)*
Daily pills are reminder of HIV in my life	Reported vs not reported	0.61 (0.51–0.72)*	1.68 (1.41–2.00)*	2.05 (1.72–2.43)*	2.27 (1.91–2.70)*
Daily pills linked to bad memories	Reported vs not reported	0.53 (0.45–0.64)*	1.99 (1.67–2.38)*	1.46 (1.22–1.75)*	3.46 (2.87–4.16)*
Worried about missing HIV medication	Reported vs not reported	0.71 (0.59–0.84)*	1.74 (1.46–2.06)*	1.69 (1.42–2.01)*	2.03 (1.71–2.41)*
Daily pills increase chance of revealing HIV status	Reported vs not reported	0.65 (0.54–0.78)*	1.90 (1.59–2.27)*	1.61 (1.34–1.94)*	3.08 (2.56–3.71)*
Ever disguised/hidden their HIV medication	Reported vs not reported	0.70 (0.58–0.83)*	1.59 (1.33–1.91)*	1.31 (1.09–1.56)*	2.26 (1.89–2.71)*
Would be stressed if someone were to see their HIV pills	Reported vs not reported	0.69 (0.58–0.82)*	2.18 (1.83–2.60)*	1.47 (1.23–1.74)*	2.60 (2.18–3.09)*
Experience of ART side effects	Non-GI only vs none	0.85 (0.66–1.09)	1.82 (1.42–2.34)*	1.47 (1.14–1.89)*	1.60 (1.25–2.05)*
	GI vs none	0.41 (0.34–0.50)*	2.99 (2.45–3.65)*	2.08 (1.69–2.56)*	3.70 (3.01–4.56)*
Mental health disorders	Reported vs not reported	0.42 (0.34–0.52)*	0.96 (0.77–1.19)	0.97 (0.78–1.20)	1.09 (0.88–1.35)
Malabsorption	Reported vs not reported	0.41 (0.22–0.78)*	2.14 (1.14–4.01)*	2.03 (0.99–4.18)	2.97 (1.44–6.13)*
Insomnia	Reported vs not reported	0.35 (0.27–0.44)*	1.72 (1.36–2.18)*	1.35 (1.06–1.73)*	1.47 (1.16–1.86)*

*ART* antiretroviral therapy, *AOR* adjusted odds ratio, *CI* confidence interval, *GI* gastrointestinal

Asterisks (*) indicate statistically significant results. All analyses were adjusted for age, gender, ethnicity, education, region, and duration of disease

^a^Optimal overall health was assessed within the past 4 weeks; self-rating of overall health as "good" or "very good" was classified as "optimal" (vs. "neither good nor poor", "poor", or "very poor")

^b^The perception that there was room for improving current HIV medication was defined as a score of ≥ 4 (on an ordinal scale from 1 to 5) in response to the question "Do you feel that there is room for improvement with your current HIV medication or do you feel that it fully meets your needs?"

^c^The feeling that "there is room for improving the way my HIV is managed" was based on a response of "Agree" or "Strongly agree" to this statement

^d^Poor self-prognosis regarding HIV mortality was an affirmative response ("Agree" or "Strongly agree") to either statement below: "HIV will reduce my life span" or "Because of my HIV, I do not plan for my old age”

### Emotional Challenges

Regarding emotional challenges to daily oral ART, 33.3% (795/2389) acknowledged that “Having to remember to take my HIV medication every day causes me stress or anxiety”, 58.4% (1394/2389) said that “Taking pills for HIV every day is a daily reminder of HIV in my life”, and 35.1% (839/2389) felt that “Taking pills for HIV every day is a link to some bad memories from my past”. Overall, 28.9% (691/2389) indicated that “Taking my HIV medication limits my day-to-day life”.

Mixed results were seen regarding the relationship between perceptions towards HIV medications and treatment satisfaction as well as other health outcomes. Self-rated health and outlook for the future were all more favorable among those who felt empowered by daily pills, e.g., those who felt that “taking my pill(s) every day reassures me that my HIV is being kept under control”. These individuals who felt empowered by daily oral dosing had 69% higher odds of optimal overall health (AOR 1.69, 95% CI 1.40–2.04) and 25% lower odds of perceiving they would die prematurely from HIV (AOR 0.75, 95% CI 0.62–0.91). Conversely, poorer health outcomes were seen among those who reported emotional challenges with daily oral dosing, including those who indicated it limits their day-to-day life, or it caused them stress. For example, those who indicated that “Having to remember to take my HIV medication every day causes me stress or anxiety” were over three times more likely to report a poor outlook in relation to their HIV-related mortality (AOR 3.30, 95% CI 2.73–4.00), compared to those without this perception.

### Psychosocial Challenges

Overall, 57.9% (1383/2389) reported ever disguising/hiding their HIV medicines to avoid sharing their HIV status. Furthermore, 45.8% of all participants (1093/2389) and 62.7% of those who ever hid their medication (867/1383) indicated that they would feel anxious or stressed if someone were to see their medication. Overall, 37.9% (906/2389) were worried that “having to take pills every day means a greater chance of revealing my HIV status to others”. Compared to those without this perception, those worried that daily pill intake increased the likelihood of unwanted disclosure were more likely to report ever disguising/hiding their HIV medicines to avoid sharing their HIV status within the past 6 months (79.1% [717/906] vs. 44.9% [666/1483], p < 0.001) or to report they would feel anxious or stressed “if someone [they] did not want to see [their] HIV pills were to find them” (68.3% [619/906] vs. 32.0% [474/1483], p < 0.001). The percentage who missed ART ≥ 1 time in the past month because they “were not in a situation where [they] felt comfortable taking [their] pills (privacy/confidentiality)” was significantly higher among those with vs. without the perception “that having to take pills every day means a greater chance of revealing my HIV status to others” (46.2% [419/906] vs. 18.5% [274/1483], p < 0.001). Overall, 29.0% of all PLHIV missed ART at least one time within the past 30 days because of privacy concerns.

Within the pooled analyses, a report of ever disguising/hiding HIV medication within the past 6 months was associated with increased odds of perceiving there was room for improving HIV medication (AOR 1.59, 95% CI 1.33–1.91), of perceiving there was room for improving their overall HIV management (AOR 1.31, 95% CI 1.09–1.56), and of having a poor outlook towards their HIV-related mortality (AOR 2.26, 95% CI 1.89–2.71). Disguising/hiding HIV medication was also inversely associated with optimal overall health (AOR 0.70, 95% CI 0.58–0.83).

Besides the impact on individual-level outcomes, privacy concerns were also associated with interpersonal relationships, including with whom HIV status was shared, as shown in Supplemental Table 1. PLHIV who were concerned that their friendships might be jeopardized if others knew of their HIV status were significantly less likely to share their HIV status with their close friends (AOR 0.75, 95% CI 0.62–0.91), close family (AOR 0.80, 95% CI 0.66–0.95), co-workers (AOR 0.47, 95% CI 0.39–0.57), wider family/circle of friends (AOR 0.58, 95% CI 0.48–0.69), or "most of the people in my life" (AOR 0.38, 95% CI 0.31–0.47). Odds of sharing HIV status with any non-HIV healthcare providers (HCPs, including family doctor or others not providing HIV care), were significantly higher among those PLHIV who were concerned about how HIV might affect their sexual relationships (AOR 1.36, 95% CI 1.10–1.68), how others might treat them differently (AOR 1.37, 95% CI 1.12–1.68), or of others spreading information about their HIV status (AOR 1.23, 95% CI 1.01–1.50). Those afraid of being prosecuted on account of HIV were less likely to share their HIV status with any non-HIV HCP (AOR 0.68, 95% CI 0.50–0.92), sexual partners (AOR 0.57, 95% CI 0.41–0.79), close family (AOR 0.60, 95% CI 0.45–0.80), or close friends (AOR 0.60, 95% CI 0.45–0.81). Those afraid of being excluded from social activities were less likely to share their HIV status with close friends (AOR 0.78, 95% CI 0.64–0.95), wider circle of family/friends (AOR 0.60, 95% CI 0.50–0.72), co-workers (AOR 0.55, 95% CI 0.45–0.67), or with most people in their life (AOR 0.45, 95% CI 0.36–0.56). Concern about job loss was associated with lower odds of sharing HIV status with wider circle of family/friends (AOR 0.74, 95% CI 0.62–0.90), co-workers (AOR 0.60, 95% CI 0.49–0.74), and most people in their life (AOR 0.58, 95% CI 0.47–0.72). Other determinants of sharing of status within social contexts are shown in Supplemental Table 1.

**Fig. 4:**
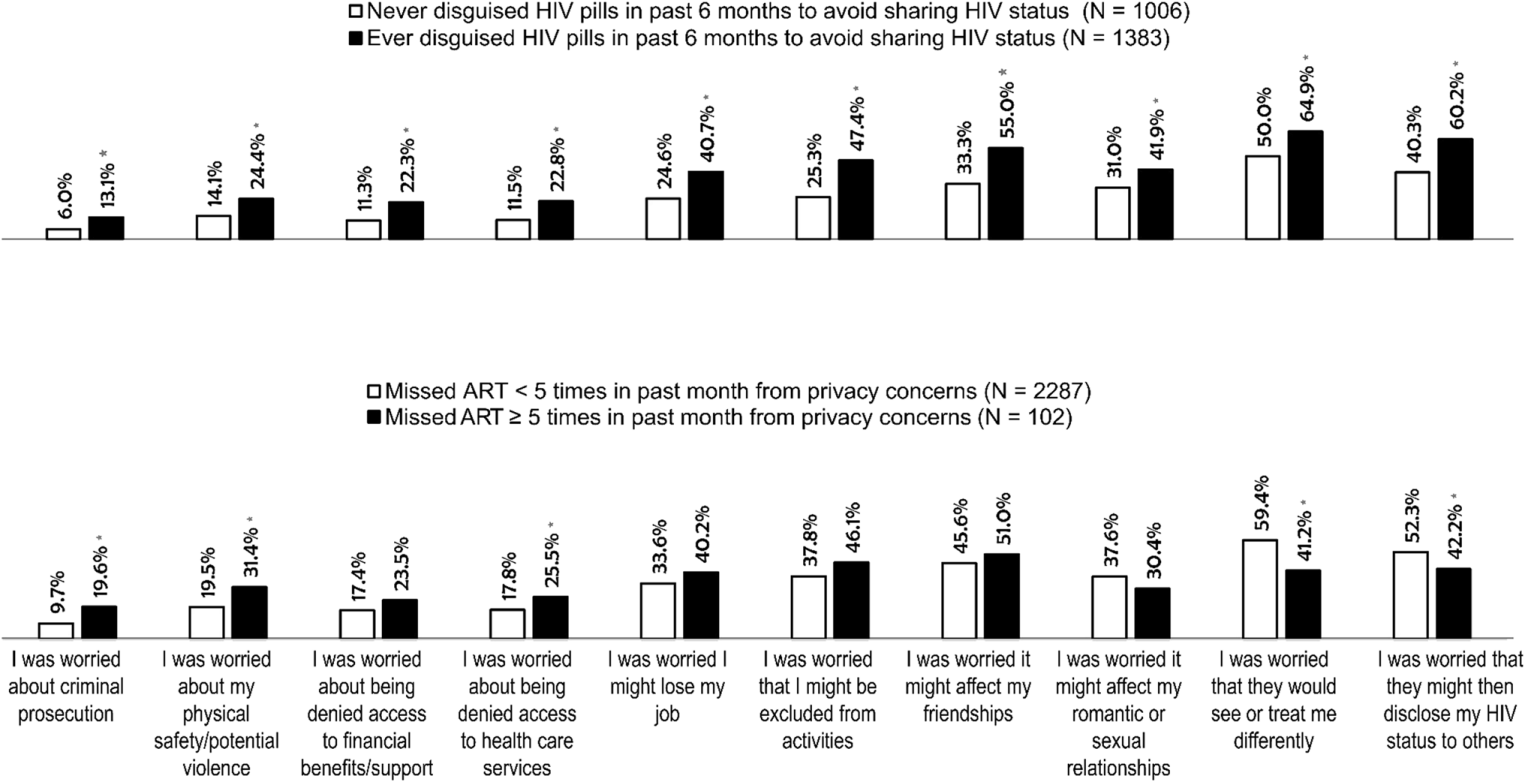
Comparison of reasons (%) for not sharing HIV status between those with or without HIV-related privacy concerns among people living with HIV from 25 countries, 2019 (N = 2389). Asterisks (*) indicate p < 0.05 based on χ^2^ tests. *ART* antiretroviral therapy

The most common privacy concerns (> 50%) among PLHIV who ever hid their HIV medication in the past 6 months revolved around their social relationships, including concerns about how knowledge of their HIV status would influence the way people saw or treated them, how individuals with whom they had shared their HIV status might tell other people, as well as how knowledge of their HIV status by others would affect their friendships (Fig. 4). PLHIV who missed ART for ≥ 5 times within the past month because of privacy concerns reported significantly higher prevalence for the following concerns than those missing for < 5 times: fear of criminal prosecution (19.6% vs. 9.7%), fear of being denied access to healthcare services (25.5% vs. 17.8%), and worry about their safety (31.4% vs. 19.5%) (all p < 0.05).

## Discussion

This study identified several physical, emotional, and psychosocial challenges faced by PLHIV in relation to daily oral ART. These challenges were associated with poor health outcomes, including perceived unmet needs towards HIV medication and overall management, and suboptimal self-rated health. The need for increased flexibility of ART delivery and improved treatment choices is underscored by our observation that while some PLHIV found daily pill taking empowering and had favorable treatment outcomes, others found it limiting and had poor outcomes.

Substance use behaviors increased with increasing duration of HIV, even after adjusting for potential confounders such as gender, geographic region, education, and ethnicity [40]. Conversely, reported experience of ART side effects declined with increasing time since diagnosis, which may suggest development of coping behaviors and mechanisms with longer duration of disease [22]. Intensified efforts are needed to screen for, and address substance use and mental health symptoms among PLHIV, given the observed high prevalence in our study population. Barrier-free access to mental health services, social support, and behavioral counseling may help increase coping skills and resilience among PLHIV [41].

Meeting the fourth “90” target of improving quality of life among PLHIV [32] calls for holistic care that considers patients’ concerns, co-morbidities, priorities, and preferences when starting or switching HIV medication to minimize the impact of HIV treatment on daily life and address unmet needs. The overall population-level impact of an unmet need is a function of both its prevalence, and its severity; these impacts of unmet needs are seldom restricted to one area of life, but rather affect multiple, overlapping aspects, including physical, psychologic, economic, and quality of life [42]. Healthcare providers can help lessen the impacts of unmet treatment needs by addressing specific concerns patients may have about ART [37]. It is encouraging to note that some PLHIV in our study were open to discussing certain HIV-related emotional challenges with their HCPs, including concerns about how HIV affected their sexual life and privacy concerns. Besides viral control, HCPs should consider such broader issues when prescribing treatment to improve adherence, retention in care, and health-related quality of life among PLHIV. The breadth of perceived unmet needs among PLHIV in our study underscores the need for alternatives in HIV therapy that would meet diverse needs and help PLHIV overcome treatment challenges. For example, long-acting (i.e., nondaily), non-oral regimens may provide a viable treatment alternative to the current exclusively daily oral regimen for patients experiencing physical, emotional, or psychosocial burdens of HIV treatment [43, 44]. Eliminating the negative cueing effect and fatigue from daily pills may help ease some of the emotional burdens identified in our study.

In line with the socio-ecological model [45], efforts to improve health-related behaviors among PLHIV must extend beyond improvements in medications or other individual-level interventions and address broader societal issues such as stigma [46]. Previous research shows that anticipated stigma may influence how patients interact with their providers [47]; for example, PLHIV in our study who were worried about HIV-related criminal prosecution were less likely to share their HIV status with HCPs other than their primary HIV care providers. Stigma within healthcare settings is an issue of urgent public health concern as it could deter PLHIV from seeking care. Unfortunately, many societies still have HIV criminalization laws or social norms that deem HIV as a taboo [48, 49], which may create a pervasive culture of secrecy among PLHIV out of fear of victimization [15–17]. Ignorance about how HIV is transmitted fuels fear of PLHIV that they might transmit HIV. These two key factors at the core of HIV-related stigma (ignorance and fear), can partly be addressed through education about Undetectable Equals Untransmittable (U = U) [38].

Improvements are also needed around patient-provider communication given previous findings that some PLHIV are hesitant raising salient treatment issues with their HCPs [37]. Quality communication can facilitate initiating discussions around alternative treatment options and allow HCPs to better understand the patient’s voice, a critical component for managing chronic diseases such as HIV [37]. Healthcare providers can lessen the impacts of treatment unmet needs by addressing specific concerns patients may have about ART. They can also provide patients with information on new treatment options that could potentially improve their quality of life [37].

### Strengths and Limitations

The strength of our study is the use of a standardized protocol to collect information from PLHIV in 25 countries; these data are recent, international in scope, and cover a breadth of topics relevant to clinical and public health practice. Nonetheless, limitations exist. First, we can only infer associations because of the cross-sectional design. Second, the findings may have limited generalizability because sampling was non-probabilistic and only a limited number of countries were included. Third, self-reports may be subject to misclassification from poor recall and other cognitive biases. This may be significant, especially that PLHIV have been shown to have some cognitive impairments [27]. Fourth, the lack of objective measure of symptoms or diagnosis may possibly result in misreporting. For example, self-reported conditions ever diagnosed within lifetime may overestimate prevalence of current health conditions. Furthermore, the conditions assessed were neither exhaustive (i.e., not all conditions assessed), nor specific (e.g., “liver disease” assessed as one broad category). Despite these limitations, this study offers a comprehensive overview of challenges faced by PLHIV, which can inform improved patient care.

## Conclusions

Many PLHIV still face numerous physical, emotional, and psychosocial challenges in relation to daily oral ART. These include physical challenges such as medical conditions that could render swallowing or remembering to take medications difficult. Emotional and psychosocial challenges include pill fatigue, cueing of negative emotions from daily dosing, and stigma. Experience of these challenges was associated with poor health outcomes, including low treatment satisfaction, self-reported virologic failure, as well as suboptimal self-rated overall health. Innovations and alternatives in HIV therapy, including long-acting, non-oral regimens, that would meet the patients at their points of need can help them rise above the barriers identified and improve their health-related quality of life. Such innovations that offer broader choice to PLHIV are therefore warranted. At the same time, population-level interventions that promote greater acceptance within health care settings and resilience-based strategies for PLHIV to combat stigma can improve health-related quality of life.

## Electronic supplementary material

Below is the link to the electronic supplementary material.

Supplementary file1 (DOCX 18 kb)
