# Correction to: Physical, Emotional, and Psychosocial Challenges Associated with Daily Dosing of HIV Medications and Their Impact on Indicators of Quality of Life: Findings from the Positive Perspectives Study

**DOI:** 10.1007/s10461-021-03206-y

**Published:** 2021-03-11

**Authors:** Patricia de los Rios, Chinyere Okoli, Erika Castellanos, Brent Allan, Benjamin Young, Garry Brough, Marvelous Muchenje, Anton Eremin, Giulio Maria Corbelli, Marta McBritton, W. David Hardy, Nicolas Van de Velde

**Affiliations:** 1 ViiV Healthcare, Quebec, Canada; 2 ViiV Healthcare, Brentford, Middlesex UK; 3 Global Action for Trans* Equality (GATE), Belize City, Belize; 4 International Council of AIDS Service Organizations (ICASO), Toronto, Canada; 5 ViiV Healthcare, Research Triangle Park, NC USA; 6 Positively UK, 345 City Road, London, UK; 7 AIDS Center Foundation, Moscow, Russia; 8 European AIDS Treatment Group, Rome, Lazio Italy; 9 Instituto Cultural Barong, São Paulo, Brazil; 10 Division of Infectious Diseases, Johns Hopkins University School of Medicine, Baltimore, MD USA

## Correction to: AIDS and Behavior (2021) 25:961–972 https://doi.org/10.1007/s10461-020-03055-1

The article Physical, Emotional, and Psychosocial Challenges Associated with Daily Dosing of HIV Medications and Their Impact on Indicators of Quality of Life: Findings from the Positive Perspectives Study, written by Patricia de los Rios · Chinyere Okoli · Erika Castellanos · Brent Allan · Benjamin Young · Garry Brough · Marvelous Muchenje · Anton Eremin · Giulio Maria Corbelli · Marta McBritton · W. David Hardy· Nicolas Van de Velde was originally published electronically on the publisher’s internet portal on 17th December 2020 without open access. With the author(s)’ decision to opt for Open Choice the copyright of the article changed on 16th February 2021 to © The Author’s 2021 and the article is forthwith distributed under a Creative Commons Attribution.

“The original article has been corrected.”

