# Supplementary material for: Physical, Emotional, and Psychosocial Challenges Associated with Daily Dosing of HIV Medications and Their Impact on Indicators of Quality of Life: Findings from the Positive Perspectives Study
Source: AIDS Behav. 2020 Oct 7;25(3):961–72. doi: 10.1007/s10461-020-03055-1 (PMC7936969; doi:10.1007/s10461-020-03055-1)
Supplement: Supplementary file 1 — Supplementary file1 (DOCX 18 kb) [file 10461_2020_3055_MOESM1_ESM.docx]

Supplemental Table 1. Adjusted odds ratios and corresponding 95% confidence intervals for the associations between specific privacy concerns and whom HIV status is shared with, among persons living with HIV in 25 countries, Positive Perspectives Survey, 2019

| Privacy concern | ↓Decreased odds of sharing HIV status with… | ↑Increased odds of sharing HIV status with… |
| --- | --- | --- |
| Worried it might affect my friendships | "Most of the people in my life" (AOR = 0.38, 95% CI = 0.31-0.47) | No significant results |
|  | Co-workers (AOR = 0.47, 95% CI = 0.39-0.57) |  |
|  | Wider family/circle of friends (AOR = 0.58, 95% CI = 0.48-0.69) |  |
|  | Close friends (AOR = 0.75, 95% CI =0.62-0.91) |  |
|  | Parents, siblings, and children (AOR = 0.80, 95% CI = 0.66-0.95) |  |
| Worried it might affect my romantic or sexual relationships | No significant results | Current family doctor/other HCP not providing HIV care (AOR = 1.36, 95% CI = 1.10-1.68) |
|  |  | Close friends (AOR = 1.56, 95% CI =1.27-1.92) |
| Worried about criminal prosecution | Spouse/sexual partner (AOR = 0.57, 95% CI = 0.41-0.79) | No significant results |
|  | Parents, siblings, and children (AOR = 0.60, 95% CI = 0.45-0.80) |  |
|  | Close friends (AOR = 0.60, 95% CI = 0.45-0.81) |  |
|  | Current family doctor/other HCP not providing HIV care (AOR = 0.68, 95% CI = 0.50-0.92) |  |
| Worried about being denied access to health care services | No significant results | Spouse/sexual partner (AOR = 1.47, 95% CI = 1.08-2.00) |
|  |  | Close friends (AOR = 1.31, 95% CI = 1.02-1.69) |
| Worried that they would see or treat me differently | "Most of the people in my life" (AOR = 0.52, 95% CI = 0.42-0.63) | Current family doctor/other HCP not providing HIV care (AOR = 1.37, 95% CI = 1.12-1.68) |
|  | Co-workers (AOR = 0.62, 95% CI = 0.51-0.76) | Close friends (AOR = 1.32, 95% CI =1.09-1.61) |
|  | Wider family/circle of friends (AOR = 0.79, 95% CI = 0.66-0.95) | Spouse/sexual partner (AOR = 1.28, 95% CI = 1.02-1.62) |
| Worried about being denied access to financial benefits/ support | No significant results | No significant results |
| Worried I might lose my job | "Most of the people in my life" (AOR = 0.58, 95% CI = 0.47-0.72) | Parents, siblings and children (AOR = 1.24, 95% CI = 1.02-1.51) |
|  | Co-workers (AOR = 0.60, 95% CI = 0.49-0.74) |  |
|  | Wider family/circle of friends (AOR = 0.74, 95% CI = 0.62-0.90) |  |
| Worried that I might be excluded from activities | "Most of the people in my life" (AOR = 0.45, 95% CI = 0.36-0.56) | No significant results |
|  | Co-workers (AOR = 0.55, 95% CI = 0.45-0.67) |  |
|  | Wider family/circle of friends (AOR = 0.60, 95% CI = 0.50-0.72) |  |
|  | Close friends (AOR = 0.78, 95% CI =0.64-0.95) |  |
| Worried that they might then disclose my HIV status to others | "Most of the people in my life" (AOR = 0.39, 95% CI = 0.32-0.48) | Spouse/sexual partner (AOR = 1.34, 95% CI = 1.06-1.68) |
|  | Wider family/circle of friends (AOR = 0.71, 95% CI = 0.59-0.85) | Current family doctor/other HCP not providing HIV care (AOR = 1.23, 95% CI = 1.01-1.50) |
|  | Co-workers (AOR = 0.53, 95% CI = 0.44-0.65) |  |
| Worried about my physical safety/potential violence | Co-workers (AOR = 0.63, 95% CI = 0.49-0.80) | No significant results |

Note: Estimates are adjusted odds ratios with accompanying 95% confidence intervals, controlling for age, gender, ethnicity, education, region, and duration of HIV.
